# Supplementary material for: Local strain inhomogeneities during electrical triggering of a metal–insulator transition revealed by X-ray microscopy
Source: Proc Natl Acad Sci U S A. 2024 Aug 15;121(34):e2317944121. doi: 10.1073/pnas.2317944121 (PMC11348337; doi:10.1073/pnas.2317944121)
Supplement: Supplementary file 1 — Appendix 01 (PDF) [file pnas.2317944121.sapp.pdf]

Local strain inhomogeneities during electrical triggering of a metal-insulator transition revealed by X-ray microscopy

Pavel Salev[1], Elliot Kisiel[2,3], Dayne Sasaki[4], Brandon Gunn[2], Wei He[2], Mingzhen Feng[4], Junjie Li[2], Nobumichi Tamura[5], Ishwor Poudyal[3], Zahir Islam[3], Yayoi Takamura[4], Alex Frano[2], Ivan K. Schuller[2]

[1]Department of Physics and Astronomy, University of Denver, Denver, CO 80210
[2]Department of Physics, University of California San Diego, La Jolla, CA 92093
[3]X-ray Science Division, Argonne National Laboratory, Argonne, IL 60439
[4]Department of Materials Science and Engineering, University of California Davis, Davis, CA 95616
[5]Advanced Light Source, Lawrence Berkeley National Laboratory, Berkeley, CA 94720

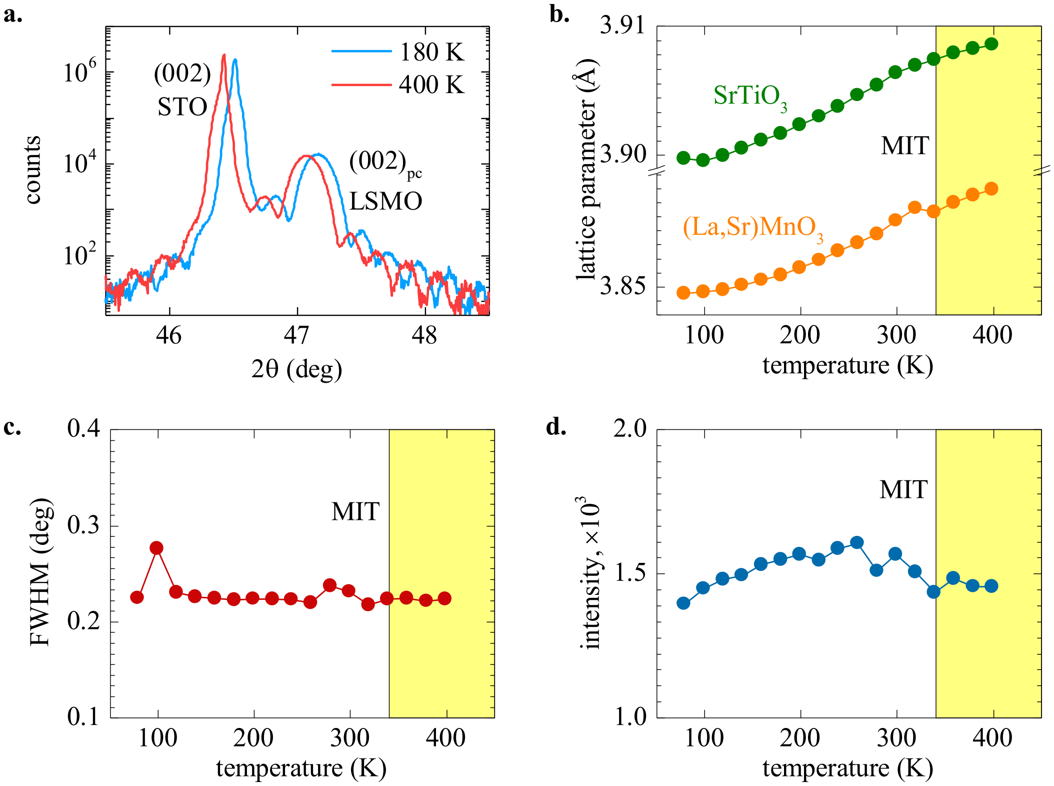

Suppl. Fig. S1. Temperature dependent x-ray diffraction measurements of an unpatterned $(La,Sr)MnO_3$ thin film sample. **a,** Diffraction pattern in the vicinity of (002) Bragg peak of the $SrTiO_3$ substrate recorded at two temperatures, 180 K (blue line) and 400 K (red line). Equilibrium thermal expansion of both film and substrate can be observed. **b,** Temperature dependence of the out-of-plane lattice constants of $SrTiO_3$ substrate (green symbols) and $(La,Sr)MnO_3$ film (orange symbols). **c, d,** Temperature dependence of the full-width-at-half-maximum (**c**) and intensity (**d**) of the $(002)_{pc}$ Bragg peak of $(La,Sr)MnO_3$. Shaded yellow regions in **b** – **c** highlight the temperature range where $(La,Sr)MnO_3$ is in the insulating phase. No pronounced structural anomalies can be observed across the metal-insulator transition either in the lattice constant, Bragg peak width or intensity. All measurements were performed in a laboratory-based x-ray system using monochromatic Cu K-α source.
